# Selective Lobar Blockade With a Bronchial Blocker in Combination With a Double Lumen Tube to Manage Refractory Hypoxemia: A Case Report

**DOI:** 10.7759/cureus.26638

**Published:** 2022-07-07

**Authors:** Jason R Muelleck, Luiz Maracaja, Thomas W Templeton

**Affiliations:** 1 Anesthesiology, Wake Forest School of Medicine, Winston-Salem, USA

**Keywords:** lobar blockade, hypoxemia, double lumen tube, bronchial blocker, thoracic anesthesia, one-lung ventilation

## Abstract

We present the case of a 66-year-old woman undergoing right robotic thoracoscopic lower lobectomy with refractory hypoxemia. After several failed attempts to improve oxygenation, we performed lobar isolation of the middle and lower lobes on the operative side utilizing a 5 Fr Arndt endobronchial blocker in combination with an *in situ* left-sided double lumen endotracheal tube. Once the bronchial blocker was in place in the right bronchus intermedius, 5 cm H_2_O of continuous positive airway pressure was applied via the tracheal lumen to the right upper lobe, significantly improving the patient’s oxygenation allowing for safe completion of the procedure.

## Introduction

Hypoxemia during thoracic surgery and one-lung ventilation in adults is common with some reports suggesting an incidence of 4-6% [1]. At this time there are a number of escalating approaches to manage hypoxemia during one-lung ventilation. In some cases, however, these methods may interfere with the surgical field, especially during robotic and/or video-assisted thoracoscopic surgery [2]. Lobar isolation using a bronchial blocker has been described in several previous reports as an approach to preserving the surgical field while at the same time allowing for the institution of continuous positive airway pressure (CPAP) with 100% oxygen to the uninvolved lobe or lobes on the operative side to manage refractory hypoxemia [3-5]. In this case report, we provide an in-depth description of both the technical as well as the physiologic aspects related to lobar isolation, utilizing a double lumen endotracheal tube, a bronchial blocker, and CPAP as one potential solution to managing ongoing hypoxemia. Written consent and Health Insurance Portability and Accountability Act authorization were obtained from the patient for the publication of this case report.

## Case presentation

A 66-year-old, American Society of Anesthesiologists (ASA) Physical Status 3, 82-kg female with chronic obstructive pulmonary disease, hypertension, hyperlipidemia, and 49-pack-year tobacco use presented for robotic right lower lobectomy and upper lobe wedge resection due to large right lower lobe non-small cell carcinoma with satellite lesions in the right upper lobe. Preoperative pulmonary function testing demonstrated low normal spirometric values (forced expiratory volume [FEV1]: 74%, forced vital capacity [FVC]: 81%, diffusing capacity for carbon monoxide [DLCO]: 75%). Preoperative vitals were: blood pressure (BP) 155/92, heart rate (HR) 101, oxygen saturation level (SpO2) 96% on room air.

**Figure 1:**
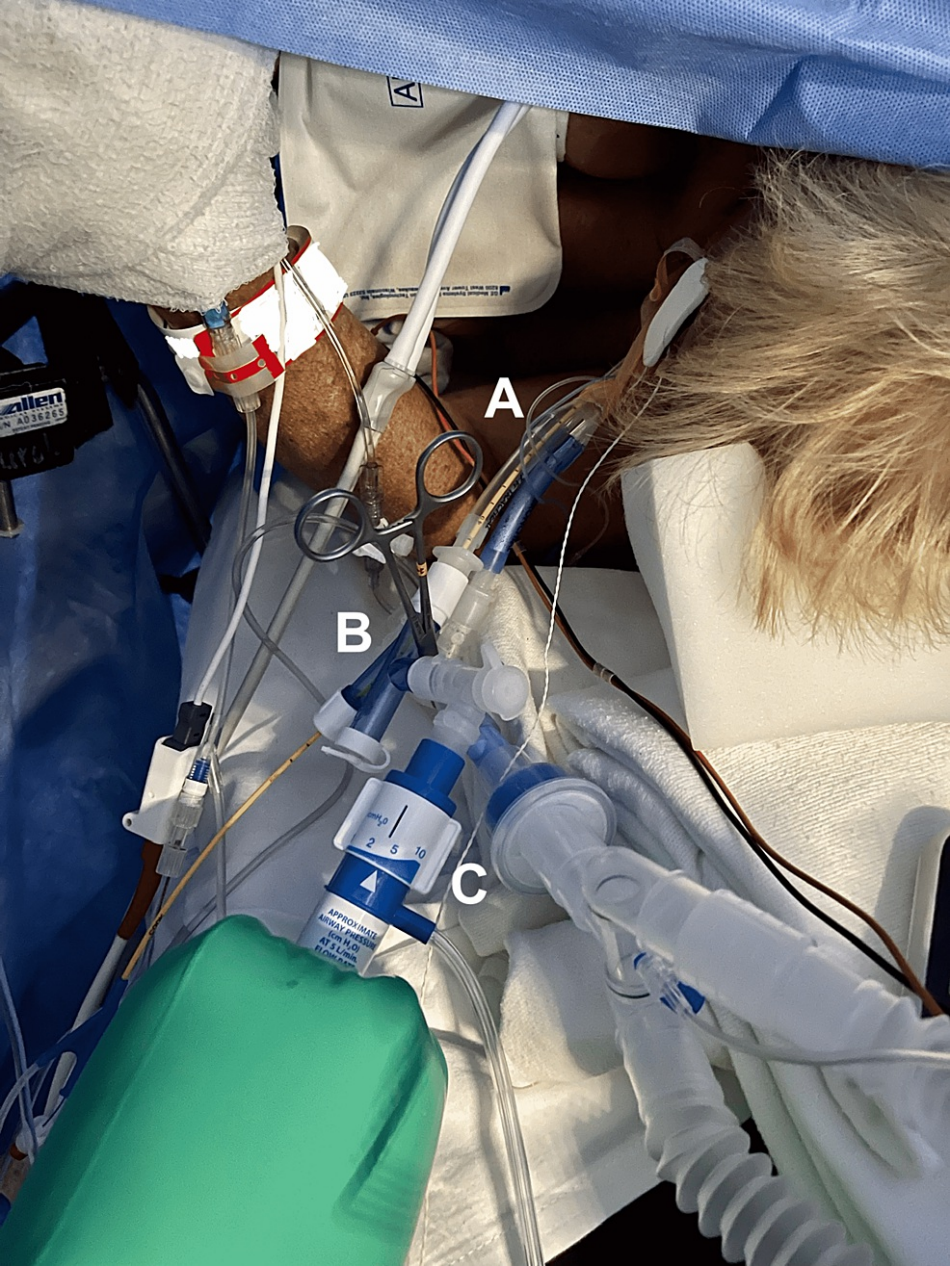
Lobar Blockade Set Up Photograph of 5 Fr Arndt bronchial blocker (A) inserted through tracheal lumen of double lumen endobronchial tube using multiport blocker adapter, (B) attached to the tracheal lumen adapter with continuous positive airway pressure applied (C).

**Figure 2:**
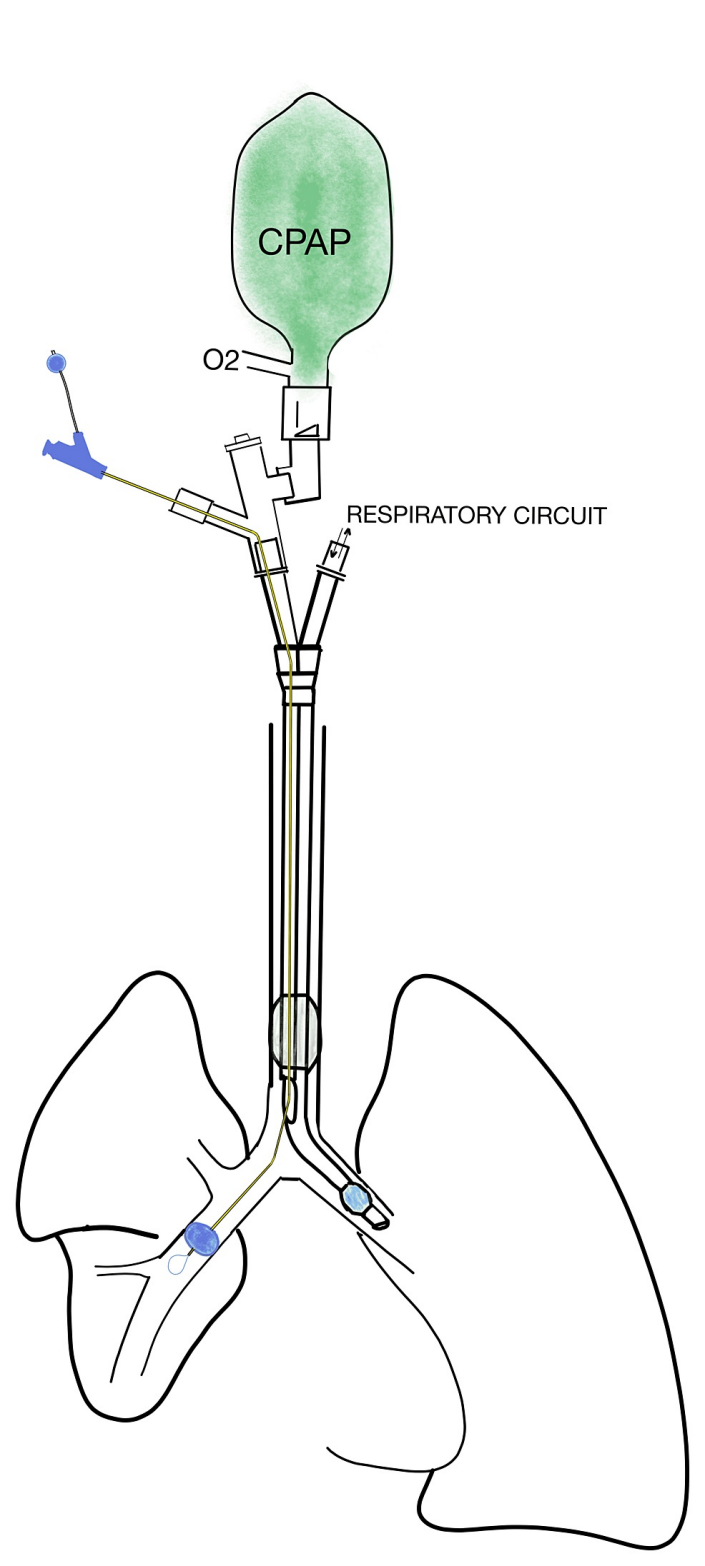
Schematic of Selective Lobar Blockade Setup with Bronchial Blocker and Double Lumen Endobronchial Tube Schematic of set up using double lumen endobronchial tube, multiport adapter, bronchial blocker, and continuous positive airway pressure device to achieve lobar blockade and apply continuous positive airway pressure to the right upper lobe.

On the day of surgery, the patient underwent three level (T3, T5, T7) ultrasound-guided right paravertebral nerve blocks with 30 mL of 0.25% bupivacaine with 1:400k epinephrine and 1.67mcg/mL clonidine (10 mL at each level). Sedation for the nerve blocks included midazolam and fentanyl, and the patient received PO acetaminophen and celecoxib for preoperative multimodal analgesia. Following induction of general anesthesia with fentanyl, ketamine, propofol, and rocuronium, a left-sided 37 Fr ShileyTM double-lumen endobronchial tube (Covidien/Medtronic, Minneapolis, MN, USA) was inserted and placement was confirmed with fiberoptic bronchoscopy. Left radial arterial line was placed for hemodynamic monitoring, however arterial blood gas was never assessed and oxygenation was monitored with SpO2. The patient was subsequently positioned in the left lateral decubitus position and prepped for robot docking. General anesthesia was maintained with isoflurane and rocuronium. One-lung ventilation (OLV) was initiated by placing a clamp on the tracheal lumen of the double lumen endotracheal tube connection. A lung protective ventilation strategy was employed utilizing tidal volumes of 4 ml/kg and positive end expiratory pressure (PEEP) of 5 cm H2O. Shortly following isolation, the SpO2 was noted to fall from 99% to 83% despite increasing the fraction of inspired oxygen (FIO2) to 100% and increasing PEEP on the dependent lung to 7 cm H2O. The patient was placed back on two-lung ventilation with improvement in the SpO2 to the high 90s. Several subsequent attempts to achieve OLV with adequate oxygen saturations continued to be unsuccessful, resulting in repeated gradual declines in SpO2 unaffected by standard tactics to troubleshoot hypoxemia during OLV including the use of 100% FIO2 and increasing PEEP to 7 cm H2O in the dependent lung. Unfortunately, the application of 5 cm H2O of CPAP on the whole surgical lung while successful in improving the patient’s oxygen saturation, significantly impacted visibility and operative conditions. Lacking other options, we felt it might be reasonable to attempt selective lobar blockade of the right middle and lower lobe with a bronchial blocker so that CPAP could be applied solely to the right upper lobe. A 5 Fr Arndt bronchial blocker (Cook Medical, Bloomington, IN, USA) was placed through the tracheal lumen using the supplied multiport adapter and was advanced into the bronchus intermedius beyond the right upper lobe take off under direct vision using a coaxial 3.7 mm flexible fiberoptic scope (Olympus Corporation of the Americas, Center Valley, PA, USA). After inflation of the occlusive balloon, 5 cm H2O of CPAP was applied via the tracheal lumen to the right upper lobe (Figures 1, 2).

Following institution of lobar blockade the patient’s SpO2 continued to remain in the mid-90s. There were no further episodes of desaturation or blocker repositioning events and surgical conditions remained optimized. At the conclusion of the procedure, the patient was returned to two-lung ventilation without issue and extubated. The remainder of her postoperative recovery was uneventful and she was discharged home on postoperative day 9.

## Discussion

Factors that correlate with hypoxemia during OLV include right-sided surgery, abnormal preoperative pulmonary function tests, low intraoperative arterial oxygen pressure during two-lung ventilation, abnormal perfusion distribution, morbid obesity, and supine positioning [6]. Application of CPAP to the surgical lung is commonly used to treat hypoxemia with OLV; however, this technique can sometimes compromise the visualization of the surgical field, especially in robotic and video-assisted surgeries. Using lobar isolation to ventilate selective lobes of the operative lung has been shown to be effective in treating hypoxemia during OLV and thoracic surgery [7-9]. However, this technique has previously been described using a bronchial blocker and single lumen endotracheal tube and not a left-sided double lumen endobronchial tube. Further, active ventilation of the other lobe on the surgical side may also impact surgical conditions. Lobar isolation using a bronchial blocker in the setting of a double lumen endobronchial tube may represent another alternative to applying CPAP to the nonoperative lobe on the operative side in cases of refractory hypoxemia in these cases.

In this case, the ShileyTM CPAP System (Covidien/Medtronic, Minneapolis, MN, USA) was utilized to apply CPAP to the right upper lobe following lobar blockade with the 5 Fr Arndt bronchial blocker. Importantly, the internal diameter (4.8 mm) of the lumens on a 35 Fr double lumen endobronchial tube are too small to accommodate both a 5 Fr bronchial blocker and an accompanying flexible fiberoptic bronchoscope. In that case, it may be necessary to use fluoroscopy to guide blocker placement or simply use a 37 Fr double lumen endobronchial tube if there are concerns for intraoperative hypoxemia during OLV from the outset.

Additionally, being able to assess bronchial anatomy remains critically important to achieving selective lobar blockade. In right-sided cases, the distance from the carina to the right upper lobe is fairly small, typically between 1.2-1.5 cm. The bronchus intermedius is only 2 cm in length. Therefore, the window of optimal positioning beyond the take-off of the right upper lobe within the bronchus intermedius is small. In left-sided cases, the clinician would need to use a right-sided double lumen endobronchial tube to achieve selective lobar blockade of the left lower lobe. Further, the proximity of the left lower lobe take-off and left upper lobe take-off may make optimal device positioning challenging [10].

Limitations to this approach include the requirement for a right-sided double lumen endobronchial tube to implement this approach in left-sided cases. Additionally, in left-sided cases, selective lobar isolation may be difficult to achieve due to challenges in placing the blocker appropriately in the longer and more narrow left-sided lobar bronchi. In right-sided cases, it may be difficult to direct the end of the bronchial blocker into the right upper lobe. Finally, even small movements can dislodge the blocker from the right upper lobe bronchus, potentially interrupting selective lobar blockade.

## Conclusions

To summarize, selective lobar blockade using a bronchial blocker through a double lumen endobronchial tube may represent an additional alternative to managing refractory hypoxemia in patients undergoing OLV and thoracic surgery. This approach can be executed even in right-sided cases when a left-sided double lumen endobronchial tube is already present. In left-sided cases the clinician will have to switch to a right-sided double lumen endobronchial tube.
